# The significance of preoperative serum carcinoembryonic antigen levels in the prediction of lymph node metastasis and prognosis in locally advanced gastric cancer: a retrospective analysis

**DOI:** 10.1186/s12876-020-01255-6

**Published:** 2020-04-10

**Authors:** Keshen Wang, Xiangyan Jiang, Yanxian Ren, Zhijian Ma, Xiaocheng Cheng, Fan Li, Jingying Xiao, Zeyuan Yu, Zuoyi Jiao

**Affiliations:** 1 Department of General Surgery, Lanzhou University Second hospital, Cheng-Guan District, Lanzhou, Gansu 730030 China; 2 Cui-ying Experimental Center, Lanzhou University Second hospital, Cheng-Guan District, Lanzhou, 730030 Gansu China

**Keywords:** Carcinoembryonic antigen, Gastric cancer, Lymph node metastasis, Prognosis, Imaging examination

## Abstract

**Background:**

In this study, we aimed to investigate the preoperative serum carcinoembryonic antigen (CEA) in the diagnosis of positive lymph node metastasis (LNM), and to evaluated the relationship between CEA and survival in patients with locally advanced gastric cancer (LAGC).

**Methods:**

The significance of the preoperative serum CEA level for the diagnose of LAGC and prediction of LNM was determined using the receiver operating characteristic (ROC) curve. The areas under the ROC of CEA were compared with those of other tumor markers or imaging examination including CT and MRI. Logistic regression was utilized to identify the risk factors predicting positive LNM. Independent prognosis factors were evaluated using univariate and multivariate COX regression analyses.

**Results:**

The ROC curves showed that the AUCs of CEA, CA199, and CA125 for diagnosing LAGC were 0.727, 0.594, and 0.566. When used to predict LNM, the AUC of CEA, CA199 and CA125 were 0.696, 0.531, and 0.588. Logistic regression analysis demonstrated that preoperative serum CEA were significantly associated with positive LNM. On combining imaging examination with CEA, the sensitivity and specificity were 85.3 and 79.4%, respectively, with the AUC equal to 0.853. The combination of CEA and imaging examination preformed the highest levels of AUC and sensitivity for diagnosing LNM, which is significantly higher than using either of them alone. Although patients with abnormal CEA have a poor prognosis, two models of multivariate analysis showed that CEA was not the independent prognosis factor for survival.

**Conclusions:**

CEA can be used to diagnose gastric cancer and determine whether it has LNM. Moreover, combined with CEA could improve the diagnostic sensitivity of imaging examination for lymph node involvement.

## Background

Gastric cancer (GC) is the fourth most common malignancy worldwide and the second leading cause of cancer-associated mortality [1]. Because most of patients are in an advanced stage at the time of diagnosis, the mortality rates for GC have continued to increase in the past decade, especially in China [2]. The reason for this may be tumour invasion or, lymph node metastasis (LNM), in addition to other factors such as differentiation, genetic mutation and patient behaviour, and postoperative recurrence and metastasis [3]. Therefore, it is important to keep looking for new prognostic factors to help in the selection of reasonable treatment strategies.

Previous studies have found that several factors have been associated with the prognosis of patients with GC, including tumour size, differentiation, lymph node metastasis (LNM) and selection of treatment. Among these factors, lymph node status may be the most reliable prognostic factor accessible [4–7]. Regional LNM is assessed via CT, MRI, or pathologic analysis, and a positive result for LNM is defined as the presence of any lymph nodes with disease. In addition, LNM is broadly recognized as an indicator of tumour progression and prognosis in GC patients following curative gastrectomy [4]. At present, D2 lymphadenectomy is the main surgery for advanced gastric cancer (AGC) as well as the majority of submucosal cancers [8]. However, it is unreasonable blindly carry out D2 lymphadenectomy in patients without LNM (N0) or with only N1 stage metastasis (N1) [9]. Therefore, accurately predicting the lymph node statue is important for selecting the optimal surgical methods preoperatively. In recent years, preoperative detection of LNM has depended on imaging studies, such as contrast-enhanced computed tomography (CECT), upper endoscopy, and magnetic resonance imaging (MRI). Nevertheless, it is very challenging for these conventional modalities to accurately detect LNM because of their low sensitivities and specificities [10, 11]. Therefore, additional methods are necessary to detect LNM before operation when imaging studies are unavailable or the results are not accurate.

Since carcinoembryonic antigen (CEA), one of the most common tumour markers, is known to reflect the clinical tumour burden in GC, it might be helpful in detecting positive lymph nodes [3, 12, 13]. However, there have been few reports on the prediction of LNM in LAGC using preoperative serum CEA. However, because early GC and LAGC are significantly different in terms of lymph node statue and survival, the predictive value of CEA for determining LNM may be low if the two stages are not separated. In addition, whether preoperative CEA levels can predict the survival of patients with GC is still controversial [11, 14–20].

The purpose of the present study was to investigate the predictive value of preoperative serum CEA for determining LNM, and to compare its sensitivity and specificity with those of imaging examination for detecting LNM, to explore the relationship between preoperative serum CEA and survival in patients with LAGC.

## Methods

### Patients

Between January 2013 and January 2018, 276 patients who were diagnosed with LAGC after surgical resection in our institution were enrolled in this retrospective study. The inclusion criteria were as follows: 1) pT2–4NxM0 resectable GC; 2) patients with histological confirmation of adenocarcinoma; 3) patients with a score of 0–2 on according to Zubrod-ECOG-WHO criteria; 4) patients with complete D2 or extend D2 lymphadenectomy; 5) patients with negative resection margins (R0); and 6) patients with complete medical records. Patients with any pretreatments, including chemotherapy and radiotherapy, were excluded. All patients were followed up via posting letters or telephone calls until death or the cut-off date (the last follow-up was 1 December 2018). All follow-up findings were collected and recorded in the database. Informed consent from the patients was waived because of the retrospective nature of this study. In addition, 172 patients with gastric polyps, chronic gastritis and gastric ulcers diagnosed by gastroscopy or pathological examination during the same period were also enrolled as the benign lesion control group. The study was approved by the Ethics Committee of the Second Hospital of Lanzhou University.

### Detection of tumor markers

Serum CEA, CA199, and CA125 concentrations were recorded from routine clinical testing. Serum CEA, CA199, and CA125 were quantitatively measured using electro chemiluminescence immunoassay (ECLIA) kits (Roche Diagnostics Gmbh, Germany). The recommended upper cut-off values for CEA, CA199, and CA125 were 3.4 ng/ml, 27 U/ml, and 35 U/ml. Testing values over the cut-off values were regarded as positive.

### Clinicopathological characteristics

The clinicopathological characteristics included age, sex, tumour location, tumour size, Lauren classification, degree of differentiation, nerve invasion, vessel invasion, tumour invasion, determination of LNM via CT or MRI, and determination of LNM via pathological were collected. Depth of tumour invasion was utilized to stage tumours according to the 7th edition UICC guidelines [21].

### Statistical analysis

Data are presented as the mean ± standard derivation for normally distributed continuous data, as the median (interquartile range, Q25 - Q75) for abnormally distributed continuous data, and as actual values for categorical data. Comparisons between two groups were performed using Student’s t test, a Wilcoxon test, or a chi-square test. The value of the three tumour markers for the diagnosis of GC and evaluation of LNM were calculated using the receiver operating characteristic (ROC) curve, which was used to distinguish the optimal cut-off value, accuracy, positive predictive value, and negative predictive value of each marker by calculating the max Youden index (sensitivity + specificity - 1). The area under the curve (AUC) was compared using the McNemar test. The potential risk factors for predicting LNM were determined by logistic regression analysis. Kaplan-Meier analysis with a log-rank test was used to calculate the overall cumulative probability, and the independent prognostic factors were identified by multivariate COX regression analysis. The primary outcome was OS, which was defined as the interval from gastrectomy to death of all causes. A *P* value less than 0.05 was considered statistically significant. All statistical analyses were conducted using Statistical Product for Social Sciences (SPSS) software (version 23.0; SPSS Inc., Chicago, USA).

## Results

**Fig. 1:**
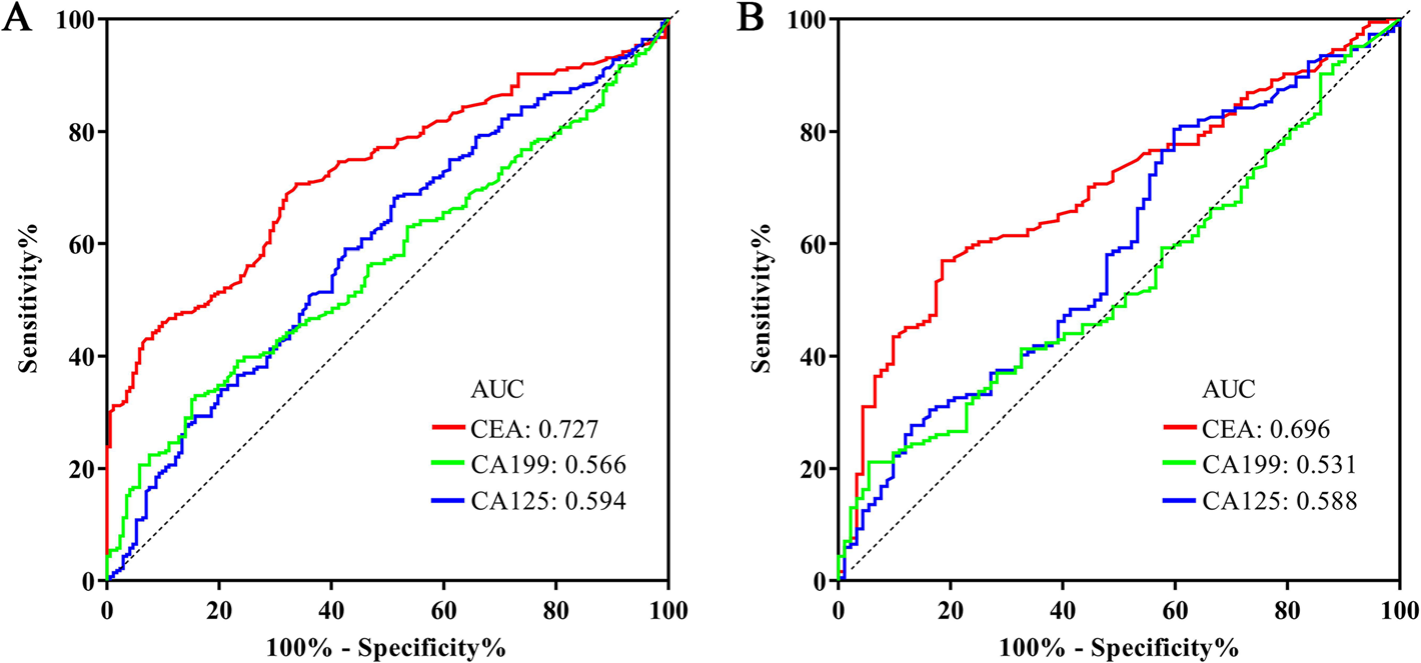
The results of ROC curve analysis for the power of CA199, CA125, and CEA in diagnosing GC and LNM. **a** ROC curve of CA199, CA125, and CEA in diagnosing GC form gastric benign lesion. **b** ROC curve of CA199, CA125, and CEA in diagnosing LNM in patients with LAGC

A total of 448 patients were included in our study, of which 276 were diagnosed with GC and 172 were diagnosed with benign gastric diseases. As shown in Table 1, the average age in the gastric cancer group was 57.28 ± 9.9 years, and this group including 203 (73.55%) males and 73 (26.45) females. The average age of patients in the benign gastric disease group was 55.74 ± 13.35 years, and this group including 97 (56.4%) males and 75 (43.6%) females. The GC group had more males, higher CEA (2.74 ng/ml, 1.685–5.62), CA199 (10.19 U/ml, 6.12–19.49), and CA125 (12.025 U/ml, 8.662–19.23) levels than the benign gastric disease group. To estimate the ability of the three tumour markers to distinguish GC from benign gastric disease, ROC curves were generated, and the results showed that the area under the curve (AUC) values for CEA, CA199, and CA 125 were 0.727(0.681–0.773), 0.594(0.54–0.648), and 0.566(0.513–0.618), respectively, with optimal cut-off values of 1.95 ng/ml, 17.12 U/ml, 9.675 U/ml (Fig. 1a, Table 2). When using the common cut-off value of 3.4 ng/ml for CEA, 27 U/ml for CA199, and 35 U/ml for CA125, the AUC values were 0.614, 0.484, and 0.4, respectively (Table 2). This finding indicated that serum CEA, CA199, and CA125 values have the ability to diagnose GC.

**Table 1:** Comparison of clinical features and tumor markers between two groups

Variables	Gastric cancer group (*n* = 276)	Benign lesion group (*n* = 172)	*P* value
Age (years)	57.28 ± 9.9	55.74 ± 13.35	0.166
Sex (Male, %)	203 (73.55%)	97 (56.4%)	0.001
CEA (ng/ml)	2.74 (1.685–5.62)	1.525 (1.09–2.385)	< 0.001
CA199 (U/ml)	10.19 (6.12–19.49)	8.95 (5.895–13.29)	0.019
CA125 (U/ml)	12.025 (8.662–19.23)	9.99 (7.447–14.8)	0.001

**Table 2:** Diagnostic performances of three serum tumor markers for differentiating GC from benign gastric disease

Group	Cut-off value	Sensitivity	Specificity	Positive predictive value	Negative predictive value	Accuracy	AUC (95%CI)
CEA	1.95 ng/ml	0.707	0.663	0.771	0.585	0.69	0.727(0.681–0.773)
	3.4 ng/ml	0.409	0.942	0.919	0.499	0.614	0.676(0.627–0.725)
CA199	17.12 U/ml	0.843	0.33	0.771	0.439	0.527	0.566(0.513–0.619)
	27 U/ml	0.199	0.942	0.846	0.423	0.484	0.571(0.517–0.624)
CA125	9.675 U/ml	0.488	0.681	0.681	0.488	0.607	0.594(0.540–0.648)
	35 U/ml	0.843	0.33	0.667	0.386	0.4	0.505(0.45–0.56)

### The value of preoperative clinical characteristics and tumor markers in predicting LNM

Since the three tumour markers showed definite value in the diagnosis of GC, we further investigated whether they had the same value in the diagnosis of lymph node involvement. The ROC curves showed that the AUC values of CEA, CA199, and CA125 were 0.696 (0.634–0.759), 0.531(0.461–0.601), and 0.588 (0.517–0.66), respectively (Fig. 1b). As shown in Table 3, LNM based on pathology assessment was associated with higher CEA (*P* < 0.001), higher CA199 (*P* = 0.001), and LNM based on imaging examination (Table 3). Multivariate logistic regression analysis was applied to determine the independent risk factors for LNM, and the results demonstrated that preoperative CEA (OR,4.86; 95%CI 2.33–10.139; *P* < 0.001) and LNM base on CT or MRI (OR, 47.81, 95%CI 16.34–139.9; *P* < 0.001) independently affected LNM (Table 3).

**Table 3:** Logistic regression analysis of preoperative factors predicting LNM based on pathology in patients with LAGC

Variables		Univariate analysis			Multivariate analysis		
		LNM -, n (%)	LNM +, n (%)	*p* value	*OR*	95%*CI*	*p* value
Gender	Male	66(71.7)	137(74.5)	0.665			
	female	26(28.3)	47(25.5)				
Age (years)	≤60	50(54.3)	113(61.4)	0.299			
	> 60	42(45.7)	71(38.6)				
Tumor location	Upper	16(17.4)	46(25)	0.146			
	Middle	50(54.3)	78(42.4)				
	lower	26(28.3)	60(32.6)				
CA199 (U/ml)	≤37	87(94.6)	147(79.9)	0.001	1		
	> 37	5(5.4)	37(20.1)		2.265	0.672–7.632	0.187
CA125 (U/ml)	≤35	90(97.8)	172(93.5)	0.152			
	> 35	2(2.2)	12(6.5)				
CEA (ng/ml)	≤3.4	76(82.6)	87(47.3)	< 0.001	1		< 0.001
	> 3.4	16(17.4)	97(52.7)		4.86	2.33–10.139	
LNM base on CT or MRI	No	88(95.7)	57(31)	< 0.001	1		< 0.001
	Yes	4(4.3)	127(69)		47.81	16.34–139.9	

### The performance of serum CEA compared with that of imaging examination in determining LNM

Based on the cut-off value of 3.4 ng/ml used commonly in the clinic, the AUC of CEA for predicting LNM in the whole cohort was 0.677. Meanwhile, the AUC of imaging examination alone, including CT and MRI, was 0.823. The combination of CEA and imaging examination predicted the highest value of positive lymph nodes, which is significantly higher than the value using either of strategy alone (Table 4). Although imaging examination combined with preoperative serum CEA level showed the highest sensitivity (0.853) and accuracy (0.833), the imaging examination showed the highest specificity (0.957) and positive predictive value (0.97) for prediction the LNM.

**Table 4:** Diagnostic efficiency of conventional methods, CEA used alone and their combined use for distinguishing lymph node metastasis from gastric cancer patients

Methods	Sensitivity	Specificity	Positive predictive value	Negative predictive value	Accuracy	AUC (95%CI)	*P value*
CEA > 3.4 ng/ml	0.527	0.826	0.858	0.466	0.627	0.677(0.612–0.742)	< 0.001^a^
Imaging examination	0.690	0.957	0.97	0.607	0.779	0.823(0.774–0.873)	< 0.001^b^
Imaging examination + CEA	0.853	0.794	0.892	0.73	0.833	0.88(0.843–0.918)	

^a^ CEA vs CEA + Imaging examination

^b^ Imaging examination vs Imaging examination + CEA

### Predictive value of CEA for LNM in subgroups

Although CEA performed well in predicting LNM in the entire population, we noted that 163 (59%) patients did not have an elevated CEA (≤ 3.4 ng/ml). Therefore, we divided patients into two groups, the CEA positive group (> 3.4 ng/ml) and CEA negative group (≤ 3.4 ng/ml), and investigated the diagnostic performance of CEA for LNM respectively in these two groups. As shown in Table 5, the AUC of CEA was 0.623(0.467–0.78) in the CEA positive group and merely 0.521(0.432–0.61) in the CEA negative group. Meanwhile, the accuracy, sensitivity and positive predictive value of CEA in prediction LNM were higher in the CEA positive group than these in the CEA negative group (accuracy, 0.611 vs 0.509; sensitivity, 0.588 vs 0.09; positive predictive value: 0.934 vs 0.889), indicating that CEA has limited predictive value for LNM in those patients with CEA negativity. We also performed further analyses that in the CEA-positive group and found that, the optimal cut-off value of CEA was 7.13 ng/ml, the corresponding sensitivity was 0.588, and the specificity was 0.75.

**Table 5:** Subgroup analysis of CEA predictive value for lymph node metastasis

Group	Optimal cut-off value	Sensitivity	Specificity	Positive predictive value	Negative predictive value	Accuracy	AUC (95%CI)
CEA > 3.4 ng/ml	7.13 ng/ml	0.588	0.75	0.934	0.231	0.611	0.623(0.467–0.78)
CEA ≤3.4 ng/ml	3.21 ng/ml	0.09	0.987	0.889	0.487	0.509	0.521(0.432–0.61)

### Survival analysis

**Fig. 2:**
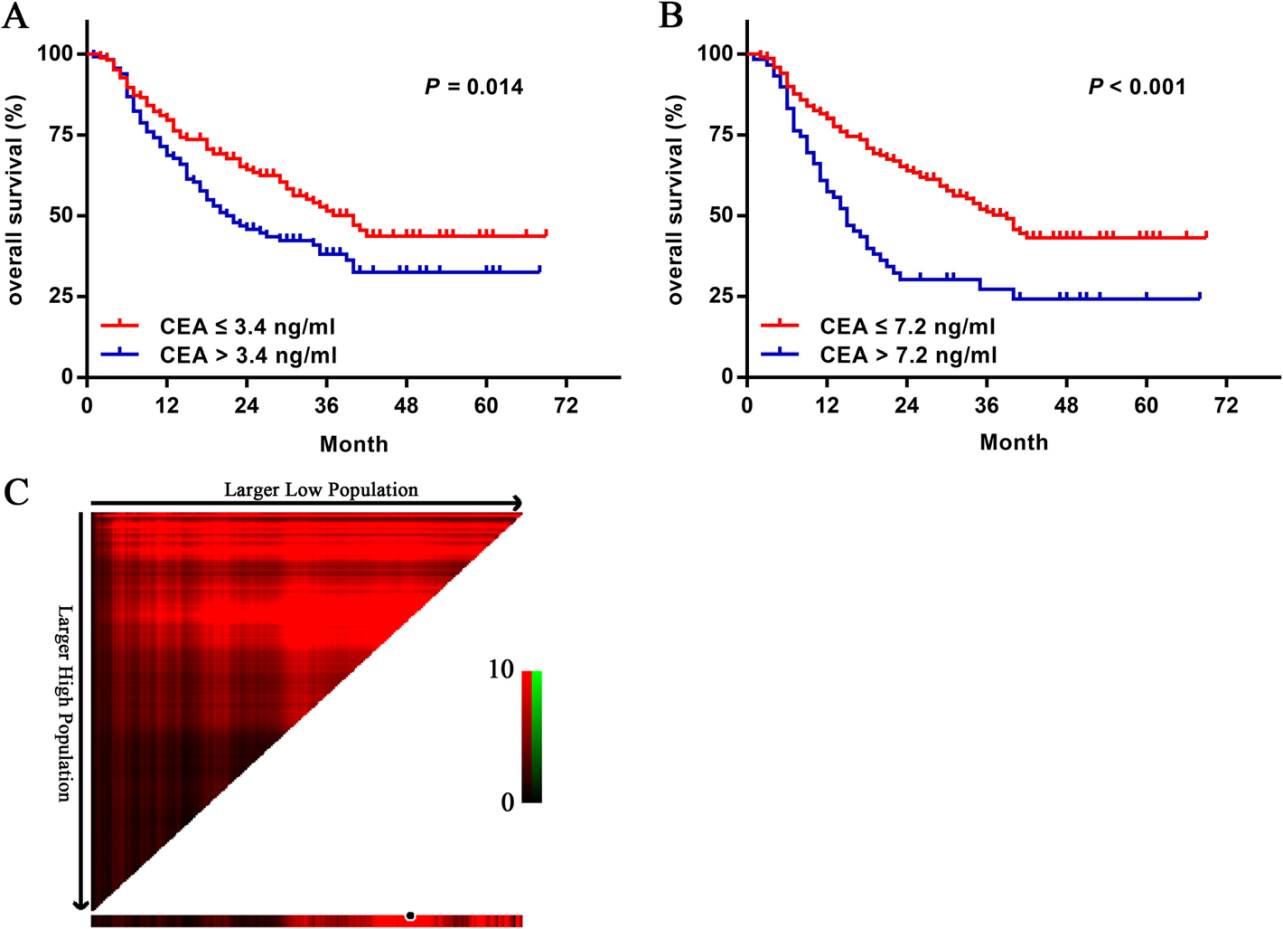
Survival analysis in 267 patients with LAGC and patients divided by X-tile plot. **a** Kaplan-Meier curves for OS when the cutoff value of CEA is 3.4 ng/ml. **b** Kaplan-Meier curves for OS when the cutoff value of CEA is 7.2 ng/ml. **c** Division of patients by the cut-off points calculated by X-tile plot

The median OS of CEA-positive group (*n* = 113) and CEA-negative group (*n* = 163) were 40 months and 21 months (*P* = 0.014), respectively (Fig. 2a). Univariate analysis showed that Lauren classification (*P* = 0.032), nerve invasion (*P* = 0.017), vessel invasion (*P* = 0.006), differentiation degree (*P* = 0.009), tumor size (*P* < 0.001), CEA (*P* = 0.016), CA199 (*P* < 0.001), pT stage (*P* < 0.001), and LNM based on pathology (*P* < 0.001) were significantly association with prognosis (Table 6). Further multivariate analysis demonstrated that preoperative CA199 level (HR, 1.608; 95%CI 1.051–2.46; *P* = 0.028), LNM based on pathology (HR:3.661, 95%CI: 2.079–6.446, *P* = 0.001) and pT stage (*P* = 0.046) were independent prognostic factors for the patients with LAGC (Table 6). The results of the X-tile plots demonstrated that the optimal cut-off point for CEA in OS prediction was 7.2 ng/ml, with a χ 2 value of 16.7, a *P* value of 0.001, and a relative risk ratio of 1:1.59 (Fig. 2b, c). Then multivariate analysis was performed again with the level of CEA stratified by 7.2 ng/ml instead of 3.4 ng/ml, and the results also showed that serum CEA was not an independent factor for OS ((Table 6).

**Table 6:** univariate and multivariate analysis for the entire patients with locally advanced gastric cancer

	Univariate analysis			^a^Multivariate analysis			^b^Multivariate analysis		
Variables	*HR*	95%CI	*p*	*HR*	95%CI	*p*	*HR*	95%CI	*p*
Gender (female)	0.857	0.584–1.258	0.431						
Age (> 60 years)	1.067	0.764–1.489	0.705						
Lauren classification			0.032			0.245			0.132
Intestinal	1			1			1		
Diffused	1.658	1.128–2.437		1.363	0.853–2.179		1.431	0.886–2.309	
Mixed	1.45	0.94–2.237		0.961	0.581–1.589		0.929	0.568–1.519	
Nerve invasion (yes)	1.675	1.094–2.51	0.017	1.098	0.633–1.903	0.74	1.068	0.612–1.863	0.816
Vessel invasion (yes)	2.07	1.228–3.487	0.006	1.008	0.505–2.01	0.983	1.102	0.555–2.191	0.781
Tumor location			0.916						
Upper	1								
Middle	0.979	0.634							
Lower	1.062	0.67–1.682							
Differentiation degree (well)	0.638	0.455–0.895	0.009	0.747	0.487–1.144	0.18	0.74	0.481–1.138	0.17
Tumor size (> 4 cm)	1.891	1.353–2.644	< 0.001	1.371	0.961–1.956	0.082	1.293	0.899–1.859	0.166
CEA (> 3.4 ng/ml)	1.507	1.081–2.102	0.016	1.097	0.75–1.604	0.632			
CEA (> 7.2 ng/ml)							1.4	0.919–2.132	0.117
CA199 (> 37 U/ml)	2.089	1.4–3.116	< 0.001	1.608	1.051–2.46	0.028	1.492	0.978–2.274	0.063
CA125 (> 35 U/ml)	1.776	0.933–3.382	0.08	1.223	0.632–2.369	0.55	1.038	0.519–2.078	0.915
LNM based on pathology (yes)	4.758	2.896–7.816	< 0.001	3.661	2.079–6.446	0.001	3.38	1.937–5.898	0.001
pT stage			< 0.001			0.04			0.074
T2	1			1			1		
T3	2.627	1.008–6.843		1.163	0.414–3.266		1.123	0.4–3.154	
T4	5.682	2.314–13.951		2.021	0.727–5.617		3.38	1.937–5.898	

^a^Using 3.4 ng/ml to stratify CEA

^b^Using 7.2 ng/ml to stratify CEA

## Discussion

Because GC is the second leading cause of cancer-related death around the world, its diagnosis and treatment are constantly attracting much attention [1]. The majority of Chinese patients with GC are at an advanced stage at the time of diagnosis, and they often have perigastric LNM involvement, which is widely considered a significant prognostic factor and a basis for treatment decisions in GC patients [4–8]. However, appropriate preoperative assessment of lymph node status is still difficult and conventional methods, such as CECT and MRI, are limited in detecting LNM due to their low sensitivity in lymph nodes less than 0.5 cm and their low sensitivity in distinguishing cancer from inflammatory hyperplasia. Thus, it is important to determine a feasible method to use in combination with conventional approaches to accurately detect preoperative LNM and develop individualized treatment protocols.

Currently, CEA, a carcino-embryonic antigen located on chromosome 19, is a commonly used tumour marker in the diagnosis of malignant tumours of the digestive tract, and high levels of CEA are closely associated with tumor burden [14, 17, 22]. Therefore, we hypothesized that CEA would be helpful for the determination of LNM in GC patients. Before we tested this, we first tested the ability of CEA to diagnose GC, because if an elevated CEA was not successful in differentiating from GC benign processes, its value for diagnosing LNM in GC would likely be limited. In this study, the median value of CEA in the GC group was 2.74 (1.685–5.62) ng/ml, which was higher than the 1.525 (1.09–2.385) ng/ml in the benign control, and the difference was statistically significant. When we used the clinically recommended 3.4 ng/ml as the cut-off value, the sensitivity was 0.409, the specificity was 0.942, and the AUC was 0.676, which are consistent with previous research [23, 24]. Therefore, we believe that the level of CEA can help diagnose GC.

In regard to determining LNM, our data show that the percentage of samples with elevated CEA, CA199, and CA125 in the LNM-positive group was significantly higher than that in the LNM-negative group. This suggests that elevated levels of CEA, CA199, and CA125 may be associated with lymph node involvement. In the multivariate regression analysis of these three tumour markers, only CEA was an independent factor in the determination of LNM. However, there have been few studies regarding the value of preoperative CEA in evaluating LNM in GC. M Ikeguchi et al. [25] reported that elevated levels of CEA before surgery were a good indicator of LNM in patients with GC. Li et al. [26] reported that preoperative serum CEA was significantly associated with positive lymph node count, and its correlation coefficient was higher than CA199 and CA724. In this study, based on the common cut-off value of 3.4 ng/ml, the sensitivity and specificity of CEA for determining LNM were 0.527 and 0.826, respectively, which were consistent with those found in a previous study [27]. To investigate the diagnostic performance of CEA, CEA levels and imaging results were first compared in our study. The results demonstrated that although the ability to determine LNM using CEA alone was significantly lower than that using imaging examination, a combination of these two methods showed the highest sensitivity, accuracy, and AUC. This indicated that CEA can assist CT or MRI to better detect lymph node status preoperatively, and added weight to show the importance of preoperative serum CEA for determining LNM. One meta-analysis reported that due to the different standard values applied by institutions, types of antibodies used, tumour stages, sensitivities and specificities, the rate of positive serum CEA results ranged from 2.3 to 60.82% [14]. It seems that the presence of CEA in the serum is dependent on increased production of CEA by cancer cells. Our data showed that 41% patients had an abnormal CEA value, so we performed a subgroup analysis to assess the performance of CEA in determining LNM in the CEA-positive group and the normal group. We found that in the CEA-positive group, the predictive power of CEA for determining LNM was strong, with the AUC was 0.623. When the cut-off value was set at 7.12 ng/ml according to the max value of the Youden index, the sensitivity was 0.588, specificity was 0.75, and accuracy was equaled to 0.611. Nevertheless, in the CEA-normal group, the AUC was merely 0.521, which indicated that when the CEA in the serum of GC patients does not increase, its ability to determine LNM is also limited. This finding needs to be validated in subsequent large sample studies.

In previous studies, it has been reported that CEA in serum is an independent prognostic factor, while CEA in tumour tissues is not [15]. Uda et al. [16] performed a retrospective study with a median follow-up of 39.6 months in 251 T2–4 GC patients. The 5-year survival rate was significantly lower in the preoperative CEA-positive group than in the CEA-negative group, and this difference was further confirmed in the multivariate analysis. In addition, the results of a meta-analysis also showed that preoperative serum CEA was an independent prognostic factor for GC [14]. However, Ucar et al. [17] reported that preoperative serum CA724, rather than CEA, was an independent factor affecting the prognosis of GC. The results of Duraker [18] also showed that preoperative serum CEA and CA199 were not independent prognostic factors for GC. In the current study, although the univariate analysis revealed that a CEA value greater than 3.4 ng/ml was associated with unfavourable survival, the findings of the multivariate analysis showed that a CEA cut-off value of 3.4 ng/ml did not allow CEA to function as an independent poor prognostic factor. Next, we also used X-plot to determine the best cut-off value of CEA for predicting prognosis, and found it to be 7.2 ng/ml. This cut-off value was consistent with the the previous literature reported [15]. We performed multivariate regression analysis with CEA cut-off value of 7.2 ng/ml instead of 3.4 ng/ml and found that CEA was still not an independent prognostic factor.

Of note, our research does have some limitations. First, this study with a retrospective nature and was performed at a single centre, which inevitably led to potential biases and a relatively small sample size. Second, the positive rate of tumour markers in GC was relatively low, which may have resulted in bias during the analysis. Third, because the patients were consecutively enrolled, some patients had a follow-up period of less than 5 years and no associated outcomes, which may had an impact on the results of the survival analysis.

## Conclusions

For patients with LAGC, preoperative CEA is a strong factor for determining LNM and may be useful for improving the sensitivity of conventional methods in determining LNM when the two approaches are combined. Serum CEA can be used for the diagnosis of GC and can assist in predicting prognosis, but it is not an independent prognostic factor.

## Data Availability

The datasets generated or analyzed during the current study are available from the corresponding author on reasonable request.

## Abbreviations

GC: Gastric cancer
LAGC: Locally advanced gastric cancer
CEA: Carcinoembryonic antigen
CA125: Carbohydrate antigen 125
CA199: Carbohydrate antigen 199
TNM: Tumor, Lymph nodes, Metastasis
CECT: Contrast-enhanced computed tomography
MRI: Magnetic resonance imaging
LNM: Lymph node metastasis
ROC: Receiver operating characteristics
AUC: Area under the ROC curve
